# Efficacy of Power Training on Sarcopenic Obesity in Community-Dwelling Older Women: A 32-Week Randomized Clinical Trial

**DOI:** 10.3390/nu17111822

**Published:** 2025-05-27

**Authors:** Luis Polo-Ferrero, Maria J. Martin, Ana Silvia Puente-González, Fausto J. Barbero-Iglesias, Susana González-Manzano, Roberto Méndez-Sánchez

**Affiliations:** 1 Department of Nursing and Physiotherapy, University of Salamanca, 37007 Salamanca, Spain; pfluis@usal.es (L.P.-F.); silviapugo@usal.es (A.S.P.-G.); fausbar@usal.es (F.J.B.-I.); ro_mendez@usal.es (R.M.-S.); 2 Institute of Biomedical Research of Salamanca (IBSAL), 37007 Salamanca, Spain; chusmt@usal.es (M.J.M.); susanagm@usal.es (S.G.-M.); 3 Department of Biochemistry and Molecular Biology, University of Salamanca, 37007 Salamanca, Spain; 4 Grupo de Investigación en Polifenoles (GIP-USAL), Unidad de Nutrición y Bromatología, University of Salamanca, 37007 Salamanca, Spain

**Keywords:** older adults, sarcopenic obesity, power training, multicomponent training, women, aging, resistance exercise, long-term, exercise intervention

## Abstract

**Background/Objectives:** Exercise is a key nonpharmacological strategy for the management of sarcopenic obesity (SO), characterized by low muscle mass and excess fat. However, long-term interventions and specific modalities, such as power training (PT), remain unexplored in this population. **Methods**: In this 32-week randomized controlled trial, 40 community-dwelling women (mean age: 77.0 ± 6.8 years) with sarcopenic obesity (per the EWGSOP2 and ESPEN criteria) were assigned to power training (PT), multicomponent training (MT), or a non-exercise control group (CG). Body composition, strength, and function were assessed pre- and post-intervention using within- and between-group analyses with effect size estimation. Nutritional intake was evaluated at baseline. **Results**: Significant pre–post intervention between-group differences were observed in appendicular skeletal muscle mass index (ASMI; *p* = 0.039), body fat percentage (BF%; *p* = 0.002), visceral fat (VF; *p* = 0.044), appendicular muscle mass (ASM; *p* = 0.021), gait speed (GS; *p* = 0.018), timed up and go test (TUG; *p* = 0.005), five-times sit-to-stand test (5STS; *p* < 0.001), and Short Physical Performance Battery (SPPB; *p* = 0.002). Large effect sizes (Cohen’s d > 0.8) were found in the PT group for all these variables. Post hoc analyses indicated that PT was significantly superior to MT in improving 5STS (*p* = 0.005) and TUG (*p* = 0.025). Notably, 35.7% of the PT participants no longer met diagnostic criteria for SO after the intervention. **Conclusions**: PT was more effective than MT and the CG in improving muscle mass, reducing fat, and enhancing functional performance in older women with SO. These findings support PT as a feasible and promising intervention.

## 1. Introduction

Sarcopenic obesity (SO) is an emerging geriatric syndrome characterized by the coexistence of excess adiposity and sarcopenia, the latter understood as the presence of reduced muscle mass and strength [1]. With aging, it is common for the loss of muscle mass and function to be accompanied by a relative or absolute increase in body fat, thus facilitating the development of SO [2,3,4]. In addition, obesity may independently contribute to muscle atrophy through adverse metabolic mechanisms associated with adipose tissue, such as oxidative stress, chronic low-grade inflammation, and insulin resistance, all of which negatively impact skeletal muscles [5]. This dual functional and metabolic burden generates a vicious cycle of metabolic dysfunction, impaired mobility, increased risk of falls and fractures, and increased morbidity and mortality in older people [6].

Currently, there is no single consensus definition of SO. Although efforts are underway to establish a global definition of sarcopenia that would allow for future diagnostic unification of SO, both entities are currently being evaluated separately following the EWGSOP2 criteria for sarcopenia and ESPEN for obesity [2,3,4]. This lack of consensus on diagnostic cut-off points hinders diagnostic standardization and complicates the accurate estimation of their prevalence [6]. In fact, a recent meta-analysis including 50 studies and a total of 86,285 older individuals reported an overall prevalence of 11%, although with a high variability according to the criteria used [7].

Despite its clinical relevance, the management of SO remains challenged by the lack of effective pharmacological treatments, making non-pharmacological interventions the cornerstone of therapy. These strategies include lifestyle modifications such as moderate caloric restriction, adequate protein intake, and regular resistance training (RT), all of which have shown efficacy in enhancing muscle mass, reducing adiposity, and improving physical function in older adults with SO [2,6,8,9]. Beyond protein intake, growing evidence highlights the role of broader nutritional factors—including macronutrient distribution, glycemic load, and total energy—as the key modulators of muscle metabolism and systemic inflammation [10]. Accordingly, careful control of nutritional intake as a potential confounding factor is crucial when designing exercise-based interventions targeting this population. Exercise, particularly RT, remains a powerful tool to address the multiple pathophysiological mechanisms associated with SO by promoting mitochondrial biogenesis, reducing systemic inflammation, and improving insulin sensitivity and muscle cell viability [8].

RT is considered an effective modality to induce muscle hypertrophy, improve strength and promote positive changes in body composition in older people [9]. However, recent studies have shown that power training (PT) could have an even greater impact on improving muscle power and functional performance in this population [11]. So far, RT and multicomponent training (MT) have been shown to be more valid strategies to improve physical function and body composition in older adults with SO, as evidenced by several meta-analyses [12,13,14]. However, many of these investigations present methodological limitations, such as short intervention durations (usually 12 weeks) and designs with high risk of bias, underscoring the need for more rigorous studies with prolonged interventions as mentioned in the meta-analyses.

Despite growing evidence on the benefits of physical exercise in older adults with SO, the specific impact of PT in this population has been poorly explored. Given the particular functional vulnerability of people with SO and the potential of PT to improve muscle power and functional performance, the key components in the prevention of disability, it is a priority to develop well-designed research evaluating the effects of PT in long-term interventions. Comparing this modality with other strategies such as MT or no intervention may provide critical evidence to optimize therapeutic recommendations and contribute to the development of more specific and effective clinical guidelines for the management of SO in older adults.

## 2. Materials and Methods

### 2.1. Study Design

A randomized clinical trial was conducted with three parallel groups: PT group, MT group, and no-exercise group (CG). The report of this clinical trial conforms to the CONSORT 2010 (Consolidated Standards of Reporting Trials) statement [15]. This trial received ethics approval from the Ethics Committee for Drug Research of the Salamanca Health Area (PI code 2023061317) on 13 June 2023. All study procedures adhered to the principles of the Declaration of Helsinki [16]. The trial was registered at ClinicalTrials.gov on 8 May 2023 (NCT06895122). This study was based on a previously published protocol describing the application of PT in older women with probable sarcopenia [17].

### 2.2. Participants

The participants were community-dwelling women aged 65 years or older who voluntarily enrolled in the geriatric revitalization program (GRP) in Salamanca. Participant eligibility was established based on diagnostic criteria for SO. Sarcopenia was defined according to the EWGSOP2 guidelines, requiring the presence of low muscle strength—assessed by handgrip strength (HG) < 16 kg or a five times sit-to-stand test (5STS) > 15 s—in combination with low muscle mass, defined as an appendicular skeletal muscle mass index (ASMI) < 5.5 kg/m^2^. Obesity was classified based on the ESPEN criteria, using either a body mass index (BMI) > 30 kg/m^2^ or a body fat percentage (BF%) > 35% [2,3]. Given the lack of a unified definition of SO, the participants were considered to have SO when both sarcopenia (EWGSOP2) and obesity (ESPEN) criteria were simultaneously met.

The exclusion criteria comprised active participation in other structured exercise programs, atrial fibrillation, uncontrolled hypertension, presence of pacemakers, recent major trauma or surgery, congenital collagen disorders, or any other clinical condition judged by the investigators to compromise safety or interfere with study participation. The participants were removed from the study if their attendance was below 85% of the sessions or if they joined another physical activity program during the intervention period. All the participants provided written informed consent before enrollment.

### 2.3. Randomization

Randomization was performed using a cluster approach, as the participants received the intervention within their assigned local centers. This design was chosen because older adults were recruited based on proximity to their residence and were already affiliated with specific community centers, making individual randomization logistically unfeasible and potentially disruptive. Group allocation was conducted using R software version 4.2.1 by an independent statistician with no involvement in data collection or intervention delivery. Allocation concealment was ensured throughout the process.

### 2.4. Blinding

Blinding was implemented at multiple levels. The participants were unaware of the specific study hypothesis and the group assignments. Outcome assessors and the data analyst were blinded to group allocation. The external researcher responsible for randomization informed only the intervention instructors of the assigned protocols, and no unblinding occurred during the study.

### 2.5. Assessments and Outcomes

A range of sociodemographic variables (personal data, number of falls in the previous year and during the intervention, and medication use), as well as body composition, strength, and functional performance, were assessed at baseline and after the 32-week intervention. Details on the standardized procedures used to ensure measurement reproducibility are provided in Table S1. All evaluations were conducted at the Research Unit of the Faculty of Nursing and Physiotherapy, University of Salamanca.

#### 2.5.1. Body Composition Variables

ASMI (ASM/height^2^), derived from appendicular skeletal muscle mass (ASM) measured via bioelectrical impedance (TANITA BC-418, Tanita Corp., Tokyo, Japan), was the primary variable used for sarcopenia diagnosis [3]. Height was measured with a stadiometer. Additional parameters included visceral fat (VF), body fat (BF), waist circumference (WC), and BMI, which were analyzed as secondary body composition outcomes.

#### 2.5.2. Strength and Functionality Variables

HG and 5STS were used as the core components for the diagnosis of confirmed sarcopenia, with HG < 16 kg and 5STS ≥ 15 s indicating low strength [3]. The Short Physical Performance Battery (SPPB), the timed up and go test (TUG), and gait speed (GS) were included to assess the severity and risk of frailty and falls [18,19,20]. The two-minute step test (TME2′) was used to measure aerobic capacity in older adults [21].

#### 2.5.3. Nutritional Assessment

Dietary intake was assessed by trained healthcare professionals before the beginning of the exercise intervention. A validated 143-item food frequency questionnaire (FFQ) from the PREDIMED study, adapted for the Spanish population [22], was used to collect data on the participants’ habitual dietary patterns over the previous year. The FFQ was semi-quantitative, providing nine frequency options ranging from “never or almost never” to “six or more times per day”, and specifying standard portion sizes for each item. Nutrient intake was calculated by multiplying the frequency of consumption by the nutrient content of each standard portion, using information from the Spanish food composition databases [23]. The nutritional analysis focused on the total energy intake, glycemic load, and the intake of macronutrients (proteins, carbohydrates, and fats), as well as specific fatty acid profiles, including monounsaturated, polyunsaturated, and saturated fatty acids. This nutritional assessment for the control of nutritional intake was used to evaluate potential confounding effects of dietary intake on the outcomes and ensure comparability across groups.

### 2.6. Interventions

The participants in the PT and MT groups completed three supervised 50 min sessions per week over a 32-week period. This duration was chosen because it aligns with the structure of the geriatric revitalization program (GRP) in Salamanca, which runs annually from September to June, thus providing a logistically feasible framework to investigate long-term effects beyond the standard 12-week protocols used in most studies. Exercise sessions were based on the ACSM guidelines and adhered to the FITT-VP principles (frequency, intensity, type, time, volume, and progression) [24]. All sessions began with active mobility and concluded with stretching and diaphragmatic breathing. The sessions were led by qualified health professionals with over two years of experience working with older adults and were conducted in senior centers across the city. Attendance was recorded to monitor adherence.

Exercise intensity in both groups was individualized and adjusted weekly by an experienced exercise therapist based on the Borg rating of perceived exertion (RPE) scale (6–20), targeting scores of 12–13 [25]. After each session, the participants reported their perceived exertion. If the RPE scores were consistently below the target range, the training load was increased.

Progression strategies were personalized and included increasing resistance, repetitions, sets, reducing rest time, or introducing unilateral movements. The researchers ensured correct technique, safety, and appropriate training load. The participants in the control group (CG) continued their usual daily routines and were advised to refrain from enrolling in new structured exercise programs during the study period to avoid potential confounding effects. Upon completion of the intervention, they were offered the opportunity to participate in the exercise program.

#### 2.6.1. Power Training (PT)

The PT protocol emphasized lower-limb strength through exercises such as squats, deadlifts, frontal and lateral lunges, leg extensions, hip extensions/abductions, and heel raises. Upper-limb exercises included wall push-ups, arm raises, elbow extensions, push-ups, and handgrip exercises. The program began with three sets of six repetitions per exercise, with load adjusted according to each participant’s RPE [25]. In the later stages, progression focused on increasing the speed of the concentric contraction phase. A detailed description of exercises, progression, and program phases is provided in the study protocol [17].

#### 2.6.2. Multicomponent Training (MT)

The MT protocol was structured into three 10 min segments focusing on aerobic, resistance, and balance components. The activities included supervised walking, lower- and upper-limb strength exercises (e.g., squats, leg extensions, wall push-ups, elbow push-ups), and both static and dynamic balance exercises. Progression criteria and detailed programming are described in the study protocol [17].

### 2.7. Sample Size

The sample size was calculated based on the ASMI, the primary outcome variable of the study. Reference was made to previous studies in older adults with SO, where the minimal detectable change in the ASM was 0.5 kg [26]. Considering the extended duration of the intervention, the higher number of sessions, and the fact that all sessions were delivered by qualified health professionals, a more demanding threshold was established, aiming to detect a larger change of 0.6 kg/m^2^ in the ASMI [27]. Assuming a common standard deviation of 0.5, a two-sided alpha level of 0.05, and a statistical power above 80%, a total of 39 participants were required to detect between-group differences across the three study arms (13 participants per group). This estimation accounts for an anticipated dropout rate of 15%.

### 2.8. Statistical Analysis

Descriptive statistics were used to summarize baseline characteristics, expressed as the means ± standard deviations for normally distributed variables, and medians with interquartile ranges for non-normally distributed variables. Normality of the data was assessed using the Shapiro–Wilk test. Between-group differences at baseline were evaluated using one-way ANOVA for parametric variables and the Kruskal–Wallis test for non-parametric variables.

To analyze within-group changes from pre- to post-intervention, paired *t*-tests were used for normally distributed variables, and Wilcoxon signed-rank tests were applied for non-parametric data. Effect sizes were calculated using Cohen’s d for parametric tests and Hedges’ g for non-parametric comparisons. Effect sizes were interpreted as small (0.2–0.4), moderate (0.5–0.7), or large (≥0.8), following Cohen’s guidelines [28].

To compare changes between the three groups (PT, MT, and CG), one-way ANOVA or the Kruskal–Wallis test was used based on the distribution of each variable. When statistically significant differences were found (*p* < 0.05), post hoc analyses were performed using Bonferroni correction for normally distributed variables and Dunn’s test for non-parametric variables to control for multiple comparisons. No additional adjustments for multiple comparisons were applied beyond Bonferroni and Dunn’s procedures.

All statistical analyses were performed using SPSS v.28 (IBM Corp., Armonk, NY, USA), with a significance level set at α = 0.05.

## 3. Results

### 3.1. Baseline Results

**Figure 1:**
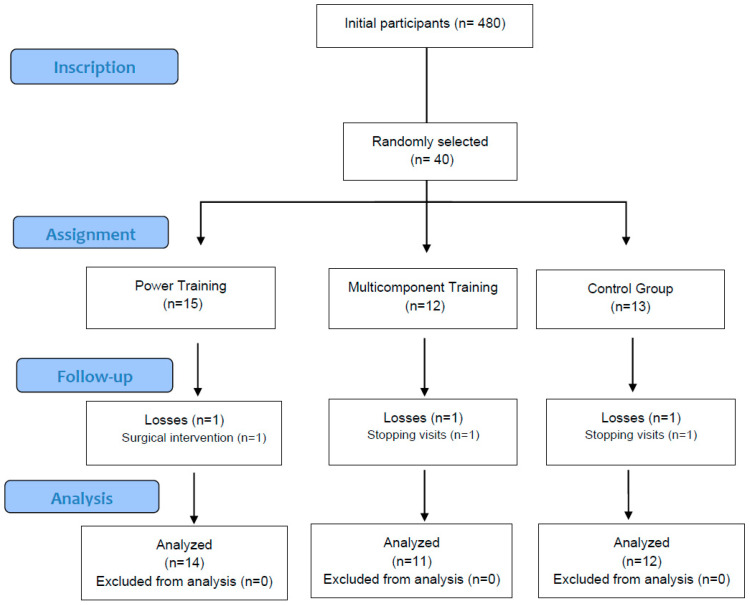
Flow diagram.

Out of the 480 participants initially screened, 40 women met the eligibility criteria and were randomly assigned to each group. The number of participants randomly assigned, who received the assigned treatment, and who were included in the analysis of the primary outcome for each group are provided. Three participants were lost to follow-up during the study, with the reasons for exclusion recorded (Figure 1). The trial was completed as intended, and no interventions were discontinued during the study.

The mean age of the participants was 76.5 ± 7.5 years, with baseline characteristics by group presented in Table 1, where descriptive statistics for each variable can be observed. Normality was assessed using the Shapiro–Wilk test, revealing that most variables did not follow a normal distribution (*p* < 0.05), except for weight, height, WC, VF, ASMI, and TME2′ (*p* > 0.05). ANOVA or Kruskal–Wallis tests showed no significant differences between the groups at baseline in anthropometric, functional, or nutritional variables (including the total energy intake, glycemic load, and macronutrient distribution), suggesting a comparable nutritional status across the groups at baseline (*p* > 0.05), confirming that all the groups were comparable prior to the intervention. Although age did not differ significantly between the groups, the observed differences in the mean age should be acknowledged, as they may carry potential relevance for interpretation. No adverse events were reported during the study, and adherence rates were comparable between the exercise groups.

### 3.2. Within-Group Analysis of Body Composition and Physical Function Post-Intervention

The PT group showed statistically significant improvements in all the body composition and functional variables (*p* < 0.05). Large effect sizes were observed in the body composition variables, including ASMI (d = 1.056), ASM (d = −0.925), WC (d = −0.886), BF% (d = −1.382), and VF (d = 0.961). Similarly, in strength and functional performance, large effect sizes were found in 5STS (d = −1.379), GS (d = −0.998), SPPB (d = −1.921), and TME2′ (d = −1.055), indicating a superior effect compared to MT and the CG.

In the MT group, significant improvements were observed in BF% (*p* = 0.026, d = −0.785), weight (*p* = 0.033, d = 0.392), and VF (*p* = 0.034, d = 0.616). Regarding functionality, improvements were found in HG (*p* = 0.042, d = −0.466), SPPB (*p* = 0.039, d = −0.546), and TME2′ (*p* = 0.026, d = −0.665); however, none reached the threshold for a large effect size.

In contrast, the CG group showed a significant reduction in weight (*p* = 0.038, d = 0.367) and BMI (*p* = 0.038, d = 0.588). Nevertheless, a decline in the 5STS performance was observed (*p* = 0.006, d = 0.939), with a large effect size, suggesting a decrease in lower-limb strength within this group. Statistical results for all the variables are presented in Table 2.

### 3.3. Comparative Group Analysis of Intervention Effects on Body Composition and Physical Function

Significant between-group differences were observed for multiple primary and secondary outcome measures. The participants in the PT group demonstrated superior improvements in the ASMI, showing a mean increase of 0.36 ± 0.34 kg/m^2^ compared to 0.10 ± 0.36 kg/m^2^ in the MT group and a decrease of −0.30 ± 1.19 kg/m^2^ in the CG (*p* = 0.039). For BF%, the PT group exhibited a significant reduction (−6.9 ± 6%), substantially greater than changes in the MT (−3.9 ± 7%) and CG (0.7 ± 4.7%) groups (*p* = 0.002). Additional significant differences were found in the VF (*p* = 0.044) and the ASM (*p* = 0.021).

Functional assessments revealed between-group differences in GS (*p* = 0.018), TUG (*p* = 0.005), 5STS (*p* < 0.001), and SPPB (*p* = 0.002). The differential effectiveness of training modalities was particularly evident in TUG (*p* = 0.025) and 5STS (*p* = 0.005), with PT producing superior gains versus MT. Complete post-intervention statistics for all the variables are presented in Table 3.

Notably, 35.7% of the participants in the PT group (*n* = 5) achieved normalization of the key parameters—muscle mass, strength, and BMI—indicating full reversal of the SO condition. This outcome highlights the clinical significance of the intervention, as no participants in the MT or CG groups met the criteria for reversal.

## 4. Discussion

The results of this randomized controlled trial provide valuable evidence on the effects of two physical training modalities, PT and MT, in community-dwelling older women with SO, a population particularly vulnerable due to the co-occurrence of excess adiposity and reduced muscle mass and strength.

Our findings confirm that PT is a superior strategy compared to both MT and the non-exercise group (CG) in improving body composition, strength, and functional performance. These results are consistent with and expand upon the conclusions of our recent meta-analysis, which demonstrated that RT significantly improves physical function and body composition in older adults with SO [13,14]. Furthermore, our outcomes align with those of a previous study evaluating high-speed circuit training, although our intervention yielded larger effect sizes (large) compared to the moderate effects reported in that study [29].

One of the most relevant findings was the significant increase in muscle mass observed in the PT group, particularly in the ASM and the ASMI, the primary outcome of this study. These improvements, with a large effect size for the ASMI (d = 1.056), exceeded those observed in the MT group and contrasted with the decline in the CG. This reinforces the effectiveness of targeted resistance interventions in preserving muscle mass in older women with SO, as emphasized in recent clinical guidelines [30]. Similar improvements were reported in previous studies [27,31], although our trial showed larger effect sizes, likely due to the longer duration, supervised progression, and intensity control. The incorporation of high-velocity contractions in PT may have played a key role in counteracting muscle loss associated with aging and obesity [32,33]. This effect may be explained by the neuromuscular benefits of emphasizing movement velocity. As shown by Balachandran et al. [29], training that prioritizes high-speed concentric contractions leads to greater improvements in muscle power and functional outcomes than traditional hypertrophy training. These adaptations are attributed to enhanced motor unit recruitment, improved synchronization, and faster firing rates, which are particularly relevant for older adults with SO who experience disproportionate declines in muscle power compared to strength. The supervised focus on movement speed by the physiotherapist may have reinforced these neural adaptations.

In terms of adiposity-related variables, PT was also effective in reducing BF% and VF, consistent with previous trials [27,34,35,36]. Skeletal muscles function as an endocrine organ by releasing myokines in response to contraction, which can influence fat metabolism, reduce inflammation, and improve insulin sensitivity. PT, by involving intense and repeated contractions of large muscle groups, may amplify this myokine response, contributing to the observed reductions in body fat and improvements in body composition [37,38]. Notably, MT also led to significant improvements in these variables, suggesting that combined aerobic, resistance, and balance training may be a viable option for improving body composition and functionality in individuals who may not tolerate or have access to more intensive protocols such as PT [12]. This supports the relevance of MT in less physically prepared individuals or those with comorbidities.

Importantly, PT also produced greater improvements in the key functional outcomes such as GS, TUG, SPPB, and 5STS. These variables are critical indicators of mobility, independence, and fall risk in older adults [3]. These findings align with results from similar studies, which have consistently demonstrated that PT enhances muscle power, a key factor in improving functional performance in older adults [27,39,40]. The substantial improvements observed in 5STS and TUG suggest that PT may be particularly effective in enhancing lower-limb power and functional capacity compared to MT. These results align with the previous literature demonstrating that PT is more strongly associated with mobility gains than traditional approaches [11]. In contrast, while MT also improved certain functional outcomes (e.g., SPPB, TME2′, and HG), the effect sizes were generally moderate and less consistent. This may be attributed to the less specific focus of MT on muscle strength, despite its value as a comprehensive approach. Meanwhile, the CG experienced a significant decline in lower-limb strength, further confirming the progressive and disabling nature of untreated SO.

These findings are partially consistent with those reported by Hui Qiu et al. who conducted a network meta-analysis comparing various exercise modalities in older adults with SO. Their results indicated that MT produced statistically significant improvements in body composition and GS, while RT was most effective for improving HG and the 30 s chair stand test [12]. However, their analysis did not include long-term studies evaluating PT as an isolated modality. In contrast, the present study isolated the effects of PT and found larger effect sizes across multiple outcomes—including the ASMI, BF%, 5STS, and SPPB—suggesting that PT may represent a particularly effective variant of RT when applied at sufficient duration and intensity in this population.

Interestingly, within the CG, a significant reduction was observed in body weight and the BMI, which might be partially explained by unmonitored factors such as changes in dietary intake, illness, or medication adjustments during the study period. However, this apparent improvement contrasts with the significant decline in the 5STS performance, with a large effect size, suggesting a clinically relevant deterioration in lower-limb strength. This decline reinforces the progressive nature of untreated SO and highlights the importance of structured exercise interventions in preventing functional decline in older adults.

Importantly, a substantial proportion of the participants in the PT group (35.7%) no longer met the diagnostic criteria for SO following the intervention. This outcome is clinically meaningful, as it reflects not only improvements in isolated parameters, but also a complete reversal of the condition in over a third of the participants. This finding underscores the potential of PT not only to alleviate the clinical manifestations of SO, but also to serve as a therapeutic strategy capable of reversing the condition when applied with adequate intensity and duration. While further studies are needed to validate these results and support the inclusion of PT in clinical practice guidelines for SO management, its unique ability to simultaneously target both muscle loss and excess adiposity positions it as a particularly promising intervention in this vulnerable population.

Despite the relevance of the findings obtained, this study has some limitations that should be considered. First, the relatively small sample size (*n* = 39), although sufficient to detect statistically significant effects, could limit the generalizability of the results to other populations. In addition, the study was conducted exclusively in older women, which restricts the applicability of the results to a male population or to more diverse contexts. Future studies with larger and more heterogeneous samples will be necessary to confirm and extend these findings. Second, complete control of lifestyle-related variables was not achieved. Although the control group was instructed to avoid participating in structured exercise programs, other potentially influential factors, such as unstructured physical activity or pharmacological changes, were not systematically monitored. Moreover, although dietary intake was assessed at baseline using a validated FFQ, no dietary monitoring was performed during the intervention. This lack of longitudinal dietary data represents a significant limitation, as unmeasured changes in eating patterns could have influenced the outcomes related to body composition and functionality, acting as potential confounders. While no significant differences were observed at baseline between the groups in the total energy intake, glycemic load, or macronutrient distribution, the absence of continuous dietary control limits our ability to fully rule out nutrition-related effects.

This study offers several important methodological and clinical strengths. It was designed as a randomized controlled trial with three parallel groups (PT, MT, and CG), incorporating blinded outcome assessments and adherence to CONSORT guidelines, which strengthens internal validity and supports causal inference. The exclusive focus on older women with SO—a high-risk and often underrepresented population in clinical research—adds to the study’s relevance. Diagnosis was based on standardized criteria (EWGSOP2 and ESPEN), ensuring consistency with international guidelines.

Notably, this is the first study to evaluate the effects of PT in older adults with SO using a long-term intervention exceeding 30 weeks. The 32-week duration clearly surpasses that of most prior investigations (typically ≤ 12 weeks), allowing for more sustained neuromuscular and metabolic adaptations to occur. All exercise sessions were supervised by qualified health professionals and tailored individually using the Borg scale, in accordance with the FITT-VP principles. This approach ensured safety, promoted adherence, and allowed for progressive overload, contributing to a low dropout rate and the overall feasibility of implementation in real-world, community settings. Notably, no serious adverse events were reported.

Finally, the intervention yielded substantial improvements, particularly in the PT group, with large effect sizes observed in the key outcomes such as the ASMI, BF%, 5STS, and SPPB. The direct comparison between PT and MT provides valuable clinical insight for optimizing exercise prescriptions in older adults with SO. Given the magnitude of improvements observed, PT should be considered a first-line therapeutic strategy for SO in older women, particularly when the clinical goal is to simultaneously improve muscle mass, reduce adiposity, and enhance functional performance.

## 5. Conclusions

This study provides compelling evidence supporting the superiority of PT over MT and a non-exercise control in improving body composition, strength, and functional performance in older women with SO. The 32-week supervised intervention resulted in clinically meaningful improvements—particularly in the ASMI, BF%, and lower-limb function—and was the first to demonstrate reversal of SO in over one-third of the participants undergoing PT. The extended duration, individualized progression, and high adherence observed reinforce the feasibility and safety of implementing structured PT programs in this high-risk population.

While MT remains a useful option for individuals with lower exercise tolerance or comorbid conditions, PT should be prioritized when the clinical objective is to restore muscle mass and enhance functionality. Given the large effect sizes and multidimensional benefits observed, PT may be considered a first-line therapeutic strategy for the management of SO in older women.

Despite these promising results, the limited sample size and exclusive inclusion of older women may restrict the generalizability of the findings. Additionally, the absence of detailed monitoring of lifestyle-related factors—such as diet and physical activity—may have influenced the outcomes. Therefore, further research involving larger and more diverse populations, as well as tighter control of potential confounders, is needed to confirm and expand upon these findings. Nevertheless, the ability of PT to concurrently address sarcopenia and excess adiposity highlights its therapeutic potential in the treatment of SO in aging populations.

## Figures and Tables

**Table 1:** Baseline characteristics of the participants by group.

	PT (*n* = 15)	MT (*n* = 12)	CG (*n* = 13)	Shapiro–Wilk Test	*p*-Value
Age	78.1 ± 6	76.9 ± 6	74.5 ± 9.5	0.886	0.443
Medication, no./day	4.4 ± 2	5 ± 3.1	3.8 ± 3.09	0.012 *	0.536 ^a^
Height, m	1.51 ± 0.08	1.51 ± 0.08	1.50 ± 0.06	0.985	0.878
ASMI, kg/m^2^	5 ± 0.45	4.9 ± 0.34	4.9 ± 0.45	0.126	0.554
BF%, %	47.8 (7.7)	46 (6)	46.7 (7.4)	<0.001 *	0.686 ^a^
Weight, kg	73.2 (21.8)	75.1 (6.6)	72.7 (14.9)	0.021 *	0.856 ^a^
WC, cm	110 ± 8.8	103.7 ± 5.3	104.9 ± 8.7	0.417	0.095
VF	15.3 ± 1.9	14.3 ± 1.2	14.2 ± 2.9	0.087	0.346
ASM, kg	10.7 (2.5)	10.8 (1.3)	10.4 (2.3)	0.009 *	0.648 ^a^
BMI, kg/m^2^	33.6 (5.2)	32.5 (3.1)	32.6 (4.8)	0.005 *	0.678 ^a^
5STS, s	18.2 (4.4)	16.9 (3.5)	17 (7)	0.012 *	0.436 ^a^
HG, kg	12.7 (3.7)	14.5 (3.6)	15.1 (6.5)	0.010 *	0.582 ^a^
GS, m/s	0.72 (0.15)	0.74 (0.31)	0.86 (0.53)	<0.001 *	0.068 ^a^
TUG, s	10.7 (6.7)	9.7 (1.4)	9.1 (4.2)	<0.001 *	0.384 ^a^
SPPB	8 (1)	8 (1.8)	9 (2)	0.005 *	0.354 ^a^
TME2′, s	81.1 ± 22.2	75.5 ± 12.9	71.1 ± 18.6	0.075	0.370
Total energy (kcal/day)	2112.2 ± 420.3	2157.0 ± 447.6	2098.7 ± 490.7	0.951	0.957
Glycemic load	108.1 ± 23.3	105.0 ± 31.5	114.3 ± 37.7	0.198	0.815
Carbohydrates (g/day)	232.8 ± 63.8	234.9 ± 61.4	224.3 ± 59.0	0.054	0.937
Proteins (g/day)	93.6 ± 17.1	94.8 ± 20.6	88.4 ± 19.7	0.937	0.774
Fats (g/day)	88.1 ± 19.3	92.8 ± 20.8	91.9 ± 27.9	0.676	0.861
Monounsaturated fatty acids (g/day)	40.3 ± 11.0	41.0 ± 6.8	41.6 ± 12.3	0.814	0.959
Polyunsaturated fatty acids (g/day)	15.9 ± 4.5	16.0 ± 8.6	14.5 ± 7.1	0.154	0.877
Saturated fatty acids (g/day)	23.7 ± 6.6	25.8 ± 8.6	24.8 ± 7.9	0.164	0.796

Note: Values expressed as the means and standard deviations for parametric variables and as the medians and interquartile ranges for non-parametric variables; *p*-values were obtained using ANOVA or the Kruskal–Wallis test, as appropriate. Glycemic load was estimated from a validated semi-quantitative FFQ. Note: * statistically significant differences; ^a^ Kruskal–Wallis test. Abbreviations: 5STS, 5 times sit-to-stand test; ASM, appendicular skeletal muscle mass; ASMI, appendicular skeletal muscle mass index; BF%, body fat percentage; BMI, body mass index; GS, gait speed; HG, hand grip strength; SPPB, Short Physical Performance Battery; TME2′, 2 min step test; TUG, timed up and go test; VF, visceral fat; WC, waist circumference.

**Table 2:** Pre–post comparisons and effect sizes for each intervention group (PT, MT, and CG), including statistical significance and magnitude of change.

	PT (*n* = 14)		MT (*n* = 11)		CG (*n* = 12)	
	*p*-Value	Cohen’s d	*p*-Value	Cohen’s d	*p*-Value	Cohen’s d
ASMI	<0.001 *	↑1.056 **	0.272	↓0.189	0.199	↓0.254
BF% ^b^	<0.001 *	↓1.382 **	0.026 *	↓0.785	0.515	↓0.146
Weight ^b^	0.015 *	↑0.617	0.033 *	↑0.392	0.038 *	↑0.367
WC	0.013 *	↓0.886 **	0.098	↑0.418	0.054	↑0.506
VF	0.007 *	↑0.961 **	0.034 *	↑0.616	0.293	↑0.162
ASM ^b^	0.001 *	↓0.925 **	0.477	↓0.151	0.093	↑0.243
BMI ^b^	0.013 *	↑0.722	0.100	↑0.544	0.038 *	↑0.588
5STS ^b^	<0.001 *	↓1.379 **	0.424	↑0.040	0.006 *	↑0.939 **
HG ^b^	0.048 *	↓0.451	0.042 *	↓0.466	0.528	↓0.018
GS ^b^	<0.001 *	↓0.998 **	0.260	↓0.129	0.906	↑0.016
TUG ^b^	0.013 *	↓0.734	0.722	↓0.108	0.086	↓0.479
SPPB ^b^	<0.001 *	↓1.921 **	0.039 *	↓0.546	0.713	↓0.122
TME2′	<0.001 *	↓1.055 **	0.026 *	↓0.665	0.155	↓0.413

Note: Within-group differences were analyzed using paired *t*-tests or Wilcoxon tests, with effect sizes reported as Cohen’s d (parametric) or Hedges’ corrections (non-parametric). Note: ^b^ non-parametric analysis; * statistically significant differences within groups; ** large effect size (d > 0.8). ↑: Increase in the value of the variable; ↓: Decrease in the value of the variable. Abbreviations: 5STS, 5 times sit-to-stand test; ASM, appendicular skeletal muscle mass; ASMI, appendicular skeletal muscle mass index; BF%, body fat percentage; BMI, body mass index; GS, gait speed; HG, hand grip strength; SPPB, Short Physical Performance Battery; TME2′, 2 min step test; TUG, timed up and go test; VF, visceral fat; WC, waist circumference.

**Table 3:** Post-intervention comparison of the mean differences across the groups, with *p*-values from ANOVA/Kruskal–Wallis tests and post hoc significance.

	PT (*n* = 14)	MT (*n* = 11)	CG (*n* = 12)	*p*-Value
**ASMI, kg/m^2^**	**0.36 ± 0.34**	**0.1 ± 0.36**	**−0.3 ± 1.19**	0.039 *
**BF%, % ^b^**	**−6.9 ± 6**	**−3.9 ± 7**	**0.7 ± 4.7**	0.002 *
Weight, kg ^b^	−5.56 ± 6.64	−2.31 ± 7.39	−1.06 ± 4.17	0.102
WC, cm	−12.4 ± 14	−6 ± 14.2	−3.6 ± 7.1	0.179
VF	−3.4 ± 3.6	−2.3 ± 3.7	0.3 ± 2.1	0.044 *
ASM, kg ^b^	0.9 ± 0.9	0.3 ± 1.6	−0.8 ± 2.9	0.021 *
BMI, kg/m^2 b^	−2.4 ± 2.9	−1 ± 3.2	−0.5 ± 1.9	0.399
5STS, s ^b^	−3.8 ± 2.6 **	−0.2 ± 3.7	1.3 ± 1.3	<0.001 *
HG ^b^	2 ± 4.3	1.9 ± 3.8	0.1 ± 4.7	0.356
GS, m/s ^b^	0.13 ± 0.1	0.03 ± 0.22	0.00 ± 0.10	0.018 *
TUG, s ^b^	−2 ± 2.5 **	0.2 ± 1.6	1 ± 1.9	0.005 *
SPPB ^b^	2.5 ± 1.2	1.3 ± 2.2	0.2 ± 1.3	0.002 *
TME2′ ^b^	10.7 ± 21.1	11.5 ± 17.2	6.3 ± 14.1	0.327

The main variables that determine sarcopenic obesity are in bold. Note: Values expressed as the differences of the means (post–pre) and standard deviations; *p*-values were obtained using ANOVA or the Kruskal–Wallis test, as appropriate. Note: ^b^ Kruskal–Wallis test; * statistically significant differences between three groups; ** statistically significant differences between PT and MT (Bonferroni’s test for normal variables and Dunn’s test for non-normal variables). Abbreviations: 5STS, 5 times sit-to-stand test; ASM, appendicular skeletal muscle mass; ASMI, appendicular skeletal muscle mass index; BF%, body fat percentage; BMI, body mass index; GS, gait speed; HG, hand grip strength; SPPB, Short Physical Performance Battery; TME2′, 2 min step test; TUG, timed up and go test; VF, visceral fat; WC, waist circumference.

## Data Availability

The datasets generated and analyzed during this study are available from the corresponding author upon reasonable request. Due to privacy protections for clinical trial participants and institutional ethics requirements, the data are not publicly available.

## Supplementary Materials

The following supporting information can be downloaded at: https://www.mdpi.com/article/10.3390/nu17111822/s1, Table S1: Standard measurements.

## Abbreviations

The following abbreviations are used in this manuscript:

5STS	5 times sit-to-stand test
ASM	Appendicular skeletal muscle mass
ASMI	Appendicular skeletal muscle mass index
BF%	Body fat percentage
BMI	Body mass index
CG	Control group
ESPEN	European Society for Clinical Nutrition and Metabolism
EWGSOP2	European Working Group on Sarcopenia in Older People 2
FFQ	Food Frequency Questionnaire
FITT-VP	Frequency, intensity, time, type, volume, progression
GS	Gait speed
HG	Hand grip strength
MT	Multicomponent training
PT	Power training
RT	Resistance training
RPE	Rating of perceived exertion
SO	Sarcopenic obesity
SPPB	Short Physical Performance Battery
TME2′	2-minute step test
TUG	Timed up and go test
VF	Visceral fat
WC	Waist circumference
