# Supplementary material for: Efficacy of Power Training on Sarcopenic Obesity in Community-Dwelling Older Women: A 32-Week Randomized Clinical Trial
Source: Nutrients. 2025 May 27;17(11):1822. doi: 10.3390/nu17111822 (PMC12157156; doi:10.3390/nu17111822)
Supplement: Supplementary file 1 [file nutrients-17-01822-s001.zip › nutrients-3658082-supplementary.pdf]

<table>
<tr><td colspan="4">Table S1. Standardized measurement of the result variables.</td></tr>
<tr><td>Body weight, ASM, ASMI, BMI, MT%, BF%, visceral fat)</td><td colspan="3">The participants' weight was adjusted (–1 kg for clothing) with confirmed hydration. Moistened electrodes and barefoot measurements ensured reliable BIA results.</td></tr>
<tr><td>WC</td><td colspan="3">Measured with a tape measure at the level of the umbilicus, after a deep breath in and out, without pressure.</td></tr>
<tr><td>5STS</td><td colspan="3">The test was conducted using a standardized armless chair (seat height: 43.2 cm). Timing began at the initial movement from the seated position and concluded upon completion of the fifth stand-to-sit transition.</td></tr>
<tr><td>HG</td><td colspan="3">Grip strength was assessed following the Southampton protocol. The participants sat upright with shoulders adducted, elbows flexed at 90°, and wrists slightly extended (0°–30°). Three consecutive measurements were taken on each hand with 10-second rest intervals, using standardized verbal encouragement. A 3-minute rest separated dominant and non-dominant hand assessments to minimize fatigue.</td></tr>
<tr><td rowspan="3">SPPB</td><td rowspan="3">The SPPB is a rapid, objective assessment comprising three functional tests, each scored from 0 to 4 points. The total scores range from 0 to 12, with higher values indicating better physical performance.</td><td>Balance</td><td>Held for 10 s with feet together, then in a semi-tandem position. The last test had to be held for more than 10 seconds in the tandem position.</td></tr>
<tr><td>Gait speed</td><td>The participants walked 4 meters at their habitual pace, with timing beginning 0.5 m before and ending 0.5 m after the measured distance to account for acceleration/deceleration. Three trials were performed, with the fastest time recorded for analysis.</td></tr>
<tr><td>5STS</td><td>Same.</td></tr>
<tr><td>TUG</td><td colspan="3">The TUG test measures the time required to stand from a chair, walk 3 meters, turn, walk back, and sit down at normal pace without physical assistance. The participants performed two trials without using their hands for support, with the faster time recorded for analysis.</td></tr>
<tr><td>TME2'</td><td colspan="3">The participants performed the test by marching in place for 2 minutes, lifting each knee to mid-point between the patella and the iliac crest. The final score reflected the total right-leg steps, meeting height criteria during the test period.</td></tr>
</table>
